# Effects of spin–orbit coupling and many-body correlations in STM transport through copper phthalocyanine

**DOI:** 10.3762/bjnano.6.254

**Published:** 2015-12-22

**Authors:** Benjamin Siegert, Andrea Donarini, Milena Grifoni

**Affiliations:** 1 Institut für Theoretische Physik, Universität Regensburg, D-93040, Germany

**Keywords:** anisotropy, copper phthalocyanine, magnetotransport, spin–orbit interaction, scanning tunneling microscopy (STM)

## Abstract

The interplay of exchange correlations and spin–orbit interaction (SOI) on the many-body spectrum of a copper phtalocyanine (CuPc) molecule and their signatures in transport are investigated. We first derive a minimal model Hamiltonian in a basis of frontier orbitals that is able to reproduce experimentally observed singlet–triplet splittings. In a second step SOI effects are included perturbatively. Major consequences of the SOI are the splitting of former degenerate levels and a magnetic anisotropy, which can be captured by an effective low-energy spin Hamiltonian. We show that scanning tunneling microscopy-based magnetoconductance measurements can yield clear signatures of both these SOI-induced effects.

## Introduction

Spin–orbit interaction (SOI) can play a major role in molecular spintronics. For example, in combination with the configuration of the non-magnetic component (organic ligand), it is known to be essential in establishing magnetic anisotropy in high-spin molecular magnets [1], and it is quite generally expected in metalorganic compounds. Effective spin-Hamiltonians are commonly used to describe this anisotropy, and usually capture well the low energy properties of these systems [1–3]. Such effective Hamiltonians have been derived microscopically for widely studied molecular magnets such as Fe_8_, Fe_4_ and Mn_12_ [4]. Recently, magnetic anisotropy effects could be directly probed by magnetotransport spectroscopy for Fe_4_ in quantum-dot setups [5–6]. An interesting question is hence if other classes of metallorganic compounds, such as the widely studied metal phthalocyanines [7–8], exhibit sizeable magnetic anisotropy induced by the interplay of electronic correlations and SOI. Indeed, in an X-ray magnetic circular dichroism (XMCD) analysis copper phthalocyanine (CuPc) was found to exhibit enormous anisotropies in both spin and orbital dipole moments [9]. Furthermore, recent experimental findings for cobalt pththalocyanine in a scanning tunneling microscopy (STM) setup [10] suggest that many-body correlations play an important role in the interpretation of the transport measurements. In a recent work [11], we have explicitly investigated long-range and short-range electron–electron correlation effects in CuPc and found a singlet–triplet splitting of the former anionic groundstate of about 18 meV, and thus a triplet as anionic ground state.

In this work we add the SOI to our analysis. We find that it further removes the triplet degeneracy by inducing splittings of few tenths of millielectronvolts. Moreover, in combination with exchange correlations, it produces a magnetic anisotropy which can in turn be captured by an effective spin Hamiltonian.

In general, the accurate calculation of the many-body properties of metallorganic molecules, such as molecular magnets or our CuPc, is a highly nontrivial task. In fact, the number of their atomic constituents is large enough that exact diagonalization is not possible and standard density-functional schemes have difficulties in capturing short ranged electron–electron correlations [4]. In order to reduce the size of the many-body Fock space, we use a basis of frontier molecular orbitals as the starting point to include electronic correlations [11–12] and construct a generalized Hubbard Hamiltonian. Furthermore, the symmetry of the molecule greatly helps to reduce the number of matrix elements one has to calculate in this basis.

To probe both SOI-induced splittings and magnetic anisotropy, we further investigated the current characteristics of a CuPc molecule in an STM configuration similar to the experiments in [13–14]: The molecule is put on a thin insulating layer grown on top of a conducting substrate. The layer functions as a tunneling barrier and decouples the molecule from the substrate. Hence the CuPc molecule acts as a molecular quantum dot weakly coupled by tunneling barriers to metallic leads (here the STM tip and the substrate). This quantum dot configuration should be favourable to experimentally probe SOI splittings and magnetic anisotropies when an external magnetic field is applied to the system, in analogy to the experiments in [6]. Indeed, we demonstrate that experimentally resolvable SOI splitting should be observed at magnetic fields of a few teslas.

The paper is organized as follows: We first derive a microscopic Hamiltonian for CuPc in the frontier orbital basis which includes exchange correlations and the SOI. This Hamiltonian is diagonalized exactly and used in further spectral analysis and transport calculations. Its spectrum is also used to benchmark the prediction of an effective spin Hamiltonian that captures well the low-energy properties of CuPc both in its neutral and anionic configurations. Finally, transport calculations with and without magnetic fields are presented and SOI-induced signatures are analyzed.

## Results and Discussion

### Microscopic model Hamiltonian for CuPc

The focus of this section is the establishment of a minimal model Hamiltonian for an isolated CuPc molecule capable to account for both electron–electron interaction and spin–orbit coupling effects. As discussed below, parameters are fixed such that experimental observations for the singlet–triplet splitting [8] as well as positions of anionic and cationic resonances [14] are satisfactorily reproduced. In its most general form and for a generic molecule such Hamiltonian reads

[1]
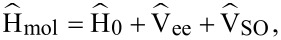


where the single-particle Hamiltonian of the molecule is given by 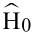, 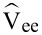 describes electronic interactions and 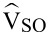 accounts for the spin–orbit interaction (SOI).

#### Single-particle Hamiltonian for CuPc

The one-body Hamiltonian 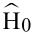, written in the atomic basis 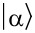, reads

**Figure 1:**
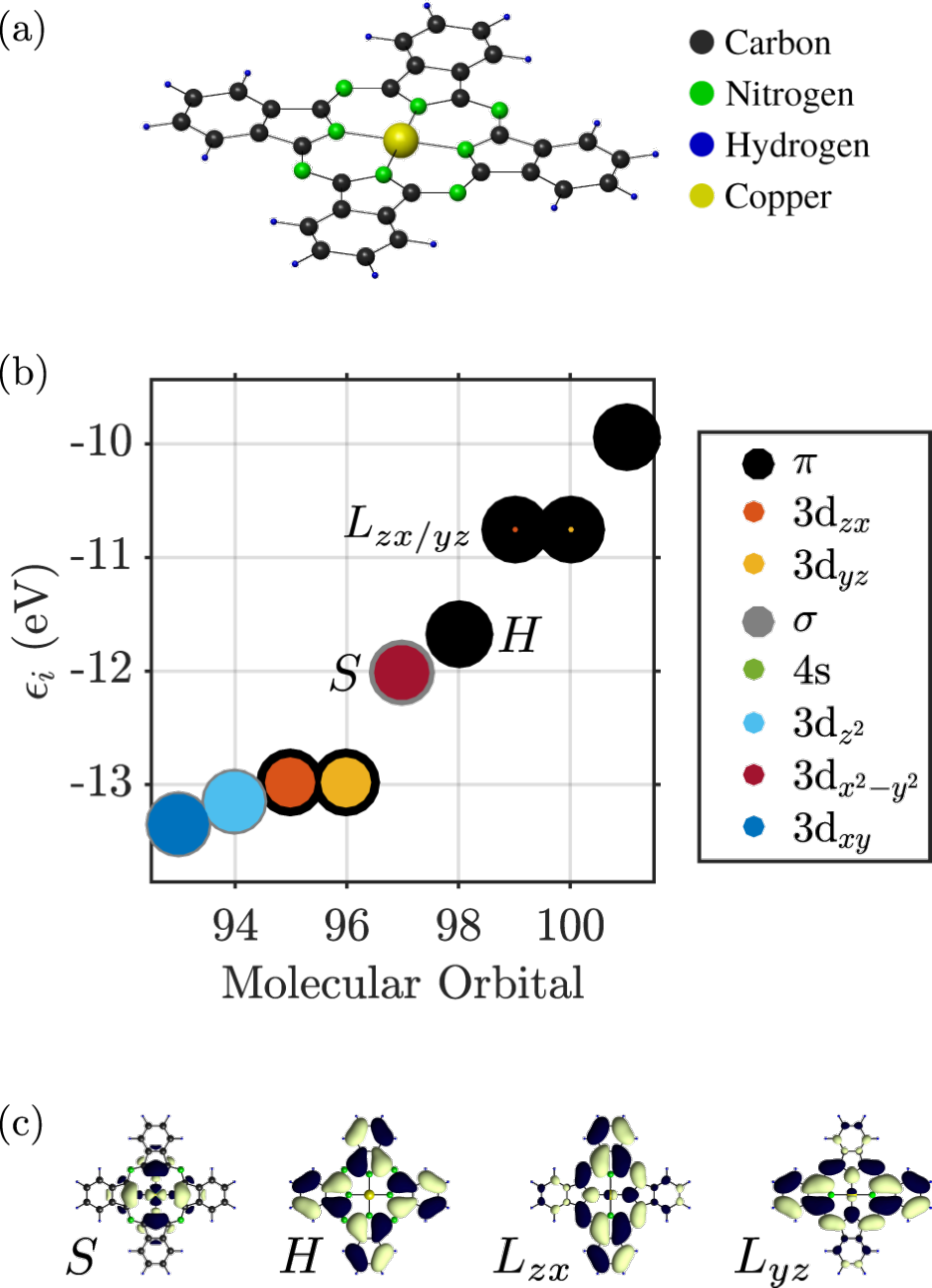
(a) Geometry and atomic composition of CuPc. (b) Single particle energies of relevant molecular orbitals. Black (grey) circles depict the π (σ) character of the corresponding orbital. The color (diameter) of the inner circles characterizes the type (weight) of the metal orbital contribution on the corresponding molecular orbital. (c) Depiction of the four frontier orbitals retained in this work: SOMO (*S*), HOMO (*H*) and LUMO*_zx/yz_* (*L_zx/yz_*).

[2]
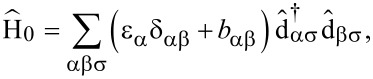


where α is a multi-index combining atomic species and orbital quantum number at position **r**_α_, see Figure 1a. For the ligand we consider the set of all 2s (1s for hydrogen), 2p*_x_* and 2p*_y_* orbitals as the σ-system, and consequently the set of 2p*_z_* orbitals as the π-system. On the metal, the 3d*_xy_*, 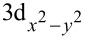, 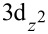 and 4s orbitals contribute to the σ-system, while the 3d*_zx_* and 3d*_yz_* belong to the π-system. This basis yields a total of 195 valence electrons for neutral CuPc. Atomic on-site energies ε_α_ and geometrical parameters were taken from [7,15]. The hopping matrix elements *b*_αβ_ in Equation 2 are obtained by using the Slater–Koster [16] and Harrison [17] LCAO schemes, similar to [18]. Numerical diagonalization of 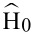 finally yields single particle energies ε*_i_*, see Figure 1b, and molecular orbitals 
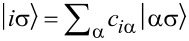
, cf. Supporting Information File 1.

Stemming from Hartree–Fock calculations for isolated atoms [15], the atomic on-site energies ε_α_ do not take into account the ionic background of the molecule and crystal field contributions. Therefore, molecular orbital energies ε*_i_* have to be renormalized with parameters δ*_i_* to counteract this shortage, yielding (cf. Supporting Information File 2)

[3]
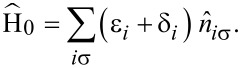


In this work we use a constant shift δ*_i_* = δ = 1.83 eV.

Due to the odd number of valence electrons, in its neutral configuration CuPc has a singly occupied molecular orbital (SOMO). When the molecule is in its anionic groundstate, this orbital does not become doubly occupied [7]. Hence, the orbitals most relevant for transport (frontier orbitals) are the SOMO (*S*), the HOMO (*H*) and the two degenerate LUMOs (*L_zx/yz_*), which transform according to the *b*_1_*_g_*, *a*_1_*_u_* and *e_g_* irreducible representations of the point group of CuPc (*D*_4_*_h_*), respectively. They are depicted in Figure 1c. The LUMO orbitals in their real-valued representations, 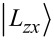 and 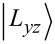, have equal contributions *c_L_* ≈ 0.097 on both 3d*_zx_* and 3d*_yz_* orbitals of the metal. Due to their degeneracy, they can be transformed into their complex, rotational invariant representations:

[4]
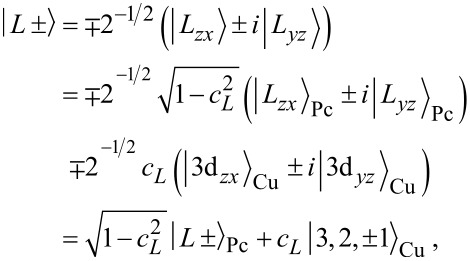


where 
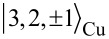
 is the *n* = 3 metal orbital with angular momentum 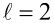 and magnetic quantum number *m* = ±1. To distinguish contributions from the pure phthalocyanine (Pc) ligand and the copper (Cu) center, we introduced 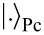 and 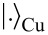, respectively. Likewise, with *c_S_* ≈ 0.90, we can write for the SOMO:

[5]
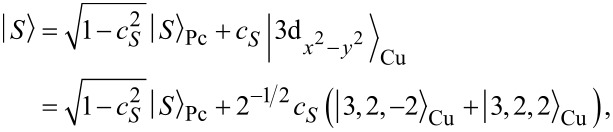


where 
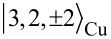
 is the *n* = 3 metal orbital with angular momentum 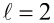 and projection *m* = *± 2* onto the *z*-axis. Finally, the HOMO has no metal contributions and thus we have trivially 
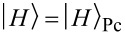
. The representations introduced in Equation 4 have the advantage that the four frontier orbitals can then be characterized by the phases φ*_i_* acquired under rotations of π/2 around the main molecular symmetry axis. For the SOMO φ*_S_* = π, for the HOMO φ*_H_* = 0 and for the two LUMOs φ*_L_*_±_ = ±π/2.

#### Many-body Hamiltonian in the frontier orbitals basis

In order to set up a minimal many-body Hamiltonian, we restrict the full Fock space to many-body states spanned by the SOMO (*S*), the HOMO (*H*) and the two LUMO (*L*±) orbitals and write Equation 1 in this basis. Hence, for neutral CuPc the number of electrons populating the frontier orbitals is *N*_0_ = 3.

We exploit the distinct phases acquired by the frontier orbitals under 90° rotations to determine selection rules for the matrix elements *V_ijkl_* in 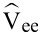,

[6]
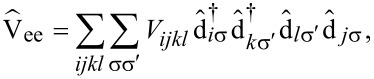


namely 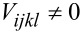 if 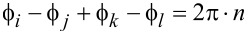, 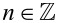, cf. Supporting Information File 2. Equation 6 in this basis then reads

[7]
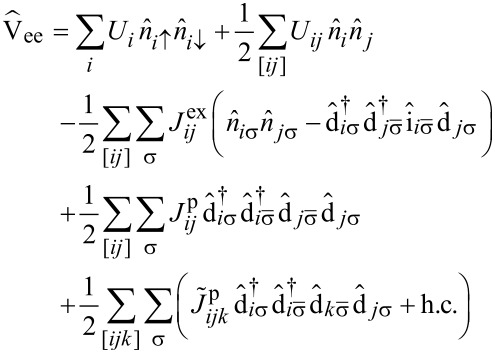


where the indices *i*, *j*, *k* now run over the set of frontier orbitals, and the notation [*ijk*] means that the sum runs only over unlike indices, i.e., *i*, *j* and *k* are different from each other in the corresponding sum. The abbreviations we introduced in Equation 7 are the orbital Coulomb interaction *U_i_* = *V_iiii_*, the inter-orbital Coulomb interaction *U_ij_* = *V_iijj_*, the exchange integral 
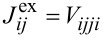
, the ordinary pair hopping term 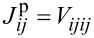 and the split pair hopping term 
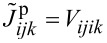
. Contributions with four different indices are found to be very small (of the order of microelectronvolts) and thus omitted in this work. The matrix elements *V_ijkl_* are calculated numerically using Monte Carlo integration [19] and renormalized with a dielectric constant ε_r_ = 2.2 in order to account for screening by frozen orbitals [12]. A table with the numerically evaluated interaction constants is found in Supporting Information File 2.

#### Spin–orbit interaction (SOI) in the frontier orbitals basis

A perturbative contribution to the bare one-body Hamiltonian 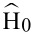 relevant in molecular systems is provided by the SOI. In the following we derive an effective spin–orbit coupling operator acting on the subset of frontier orbitals. The atomic SOI operator reads

[8]
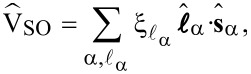


where α and 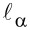 run over all atoms and shells, respectively. By evaluating Equation 8 only on the central copper atom, i.e., 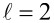 and α = Cu, 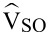 in second quantization is given by

[9]
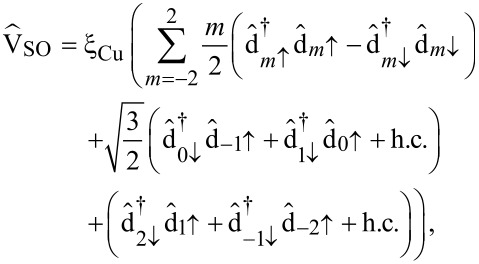


where 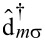 creates an electron with spin σ on the copper atom in the orbital specified by 
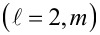
. For an electron in the 3d shell of Cu we use ξ_Cu_ ≈ 100 meV [20]. Projecting Equation 9 onto the minimal set of frontier orbitals then yields:

[10]
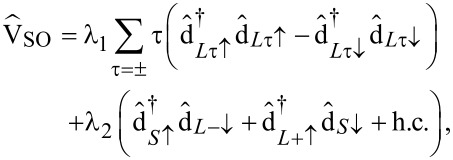


where λ_1_ = 1/2ξ_Cu_|*c_L_*|^2^ = 0.47 meV and λ_2_ = ξ_Cu_(*c_S_c_L_*)/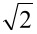 = 6.16 meV are now effective spin–orbit coupling constants. A similar analysis of SOI in CuPc, laying more focus on the central Cu atom, can be found in [21].

Finally, many-body eigenenergies *E_Nk_* and eigenstates 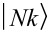, labeled after particle number *N* and state index *k*, are obtained by exact numerical diagonalization of 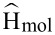 in the frontier orbitals basis. Despite numerically tractable, the problem described by 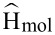 is still highly intricate, as the Fock space has dimension 4^4^ = 256. In reality, though, only few low-lying many-body states are relevant at low energies. This enables further simplification and even an analytical treatment, as discussed in the next subsection.

### Low-energy spectrum of CuPc and effective spin Hamiltonian

In the following we will analyze the neutral and anionic low-energy part of the many-body spectrum of CuPc and establish an effective Hamiltonian which enables us to analyze the low-energy behaviour in a more lucid way. To this extent, we start by observing that 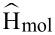 (in the considered particle number subblocks) contains different energy scales, in particular, *U > J >* λ, which suggests a hierarchy of steps. We use *U*, *J* and λ to denote the set of all Hubbard-like parameters (*U_i_*, *U_ij_*), all exchange parameters (
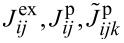
) and all SOI parameters (λ*_i_*), respectively. As a first step we set both the exchange (*J*) and SOI (λ) contributions to 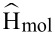 to zero and determine the neutral and anionic groundstates. In a second and third step exchange and SOI are added, respectively.

#### Neutral low-energy spectrum

In the neutral low-energy part of the spectrum, we retain the two spin-degenerate groundstates of 
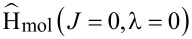
,

[11]
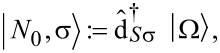


with corresponding energy 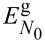. We defined 
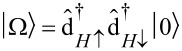
. The groundstates in Equation 11 are neither affected by 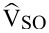 nor by the exchange terms in Equation 7. Trivially, the effective Hamiltonian in the basis of 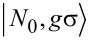 reads:

[12]
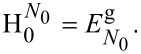


In principle Equation 7 also contains terms that act on the neutral groundstate, such as for example pair hopping terms proportional to 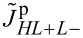, and cause admixtures with other many-body states. However, according to our full numerical calculations, these admixtures are rather small and do not affect transitions between neutral and anionic states.

#### Anionic low-energy spectrum

Continuing with the anionic low-energy part of the spectrum of 
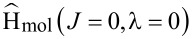
, we find an eightfold degenerate groundstate:

[13]
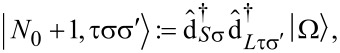


with corresponding energy 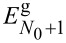. The eightfold degeneracy comes from the two unpaired spins in either SOMO or LUMO and the orbital degeneracy of the LUMO orbitals. In order to make the anionic eigenstates also eigenstates of the spin operators 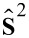 and 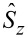, they can be rewritten as

[14]
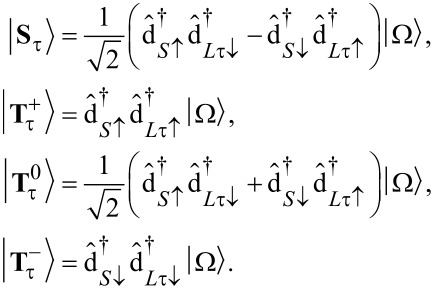


**Figure 2:**
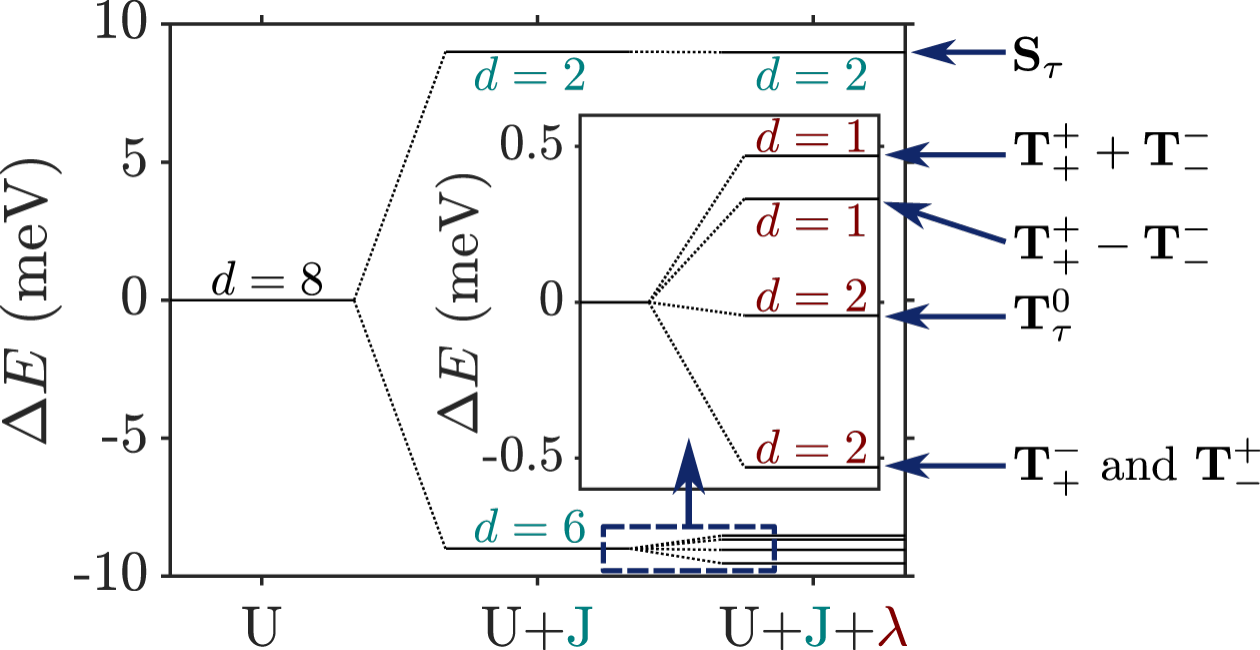
Lowest lying anionic states of CuPc, together with their grade of degeneracy *d*. Without exchange and SOI, the anionic groundstate is eightfold degenerate. When exchange interaction between SOMO and LUMOs is introduced, the degeneracy is lifted, yielding two triplets and two singlets because of the orbital degeneracy of the LUMO. SOI further splits the triplet states, generating a twofold degenerate anionic groundstate consisting of the states 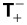 and 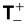.

The orbital degeneracy of the LUMOs, expressed by the index τ, is responsible for the two sets of singlets (total spin *S* = 0) and triplets (total spin *S* = 1). Considering exchange interaction in a second step, we find that only the 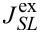 term in Equation 7,

[15]
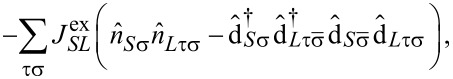


directly determines the low-energy structure of the anionic low-energy part because of the singly occupied SOMO and LUMOs: The degeneracy between singlets and triplets is lifted, see Figure 2, and we obtain

[16]
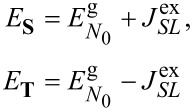


for the singlets and triplets, respectively.

Finally, to analyze in a third step how 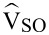 affects the low-energy part of the anionic part of the spectrum, in particular which degeneracies are lifted, we treat it as a perturbation and apply second-order perturbation theory to obtain the energy shifts. To this end, some additional states have to be considered. They are listed in Supporting Information File 3.

The states 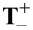 and 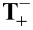 experience a downshift due to 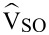 and become the groundstates. Measuring energies with respect to 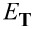, we get

[17]
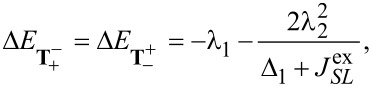


see Figure 2. Note that in our numerical calculations 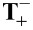 and 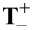 are mixed and the degeneracy of the resulting states is lifted by a small shift in the range of some μeV. A more detailed discussion concerning the mixing of 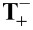 and 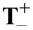 can be found in Supporting Information File 3. The next states are 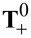 and 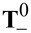 with

[18]
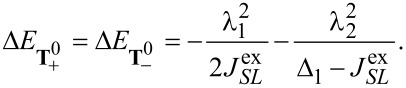


Due to their quadratic dependence on λ_1_ and λ_2_, these states change very little with 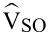. The degeneracy of the states 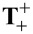 and 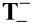 is lifted by the mixing of these states through 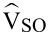. We find

[19]
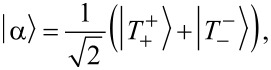


[20]
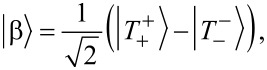


where for 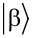 we omitted smaller additional contributions from other states. The energies change according to

[21]
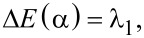


[22]
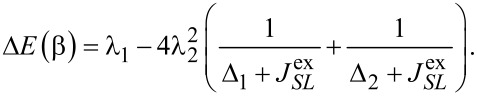


For further details we refer to Supporting Information File 3. Finally, the singlets **S**_+_ and **S**_−_, similar to 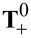 and 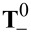, change very little (with respect to 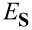):

[23]
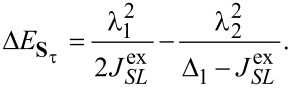


By introducing 
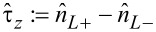
, an approximate Hamiltonian up to first order in 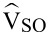 can be given for the *N*_0_ + 1 particle subblock:

[24]
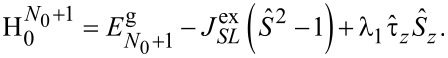


Equation 24 is one major result of this work. It shows that, similar to the well-studied molecular magnets [3–6], the interplay of spin–orbit coupling and exchange interactions yield magnetic anisotropies that can be captured by effective spin Hamiltonians. Noticeably, because Equation 24 was derived from the microscopic molecular Hamiltonian 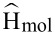, it was possible to check that deviations are in range of microelectronvolts and only of quantitative nature by comparison of the spectrum to the numerically evaluated one. Another source of magnetic anisotropy is the Dzyaloshinskii–Moriya interaction [22–23]. Although the latter is also linear with respect to the SOI, it does not appear in our model. The fundamental reason for neglecting it is the large ratio between the hopping integrals (of the order of electronvolts) and the SOI (ξ_Cu_ ≈ 100 meV), which also justifies our perturbative analysis in terms of molecular orbitals. However, for molecular quantum dots with comparable SOI and hopping integrals the Dzyaloshinskii–Moriya interaction is sizeable and produces interesting effects on magnetization [24] and transport characteristics [25].

### Interaction with magnetic fields

An experimentally accessible way to probe magnetic anisotropies is to apply external magnetic fields. In order to account for interactions of orbitals with magnetic fields, the atomic hopping matrix elements *b*_αβ_ in Equation 2 have to be corrected with Peierls phase factors,

[25]
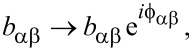


where, using the gauge 
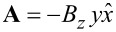
, the phase is given by

[26]
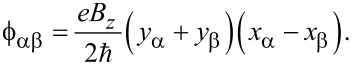


**Figure 3:**
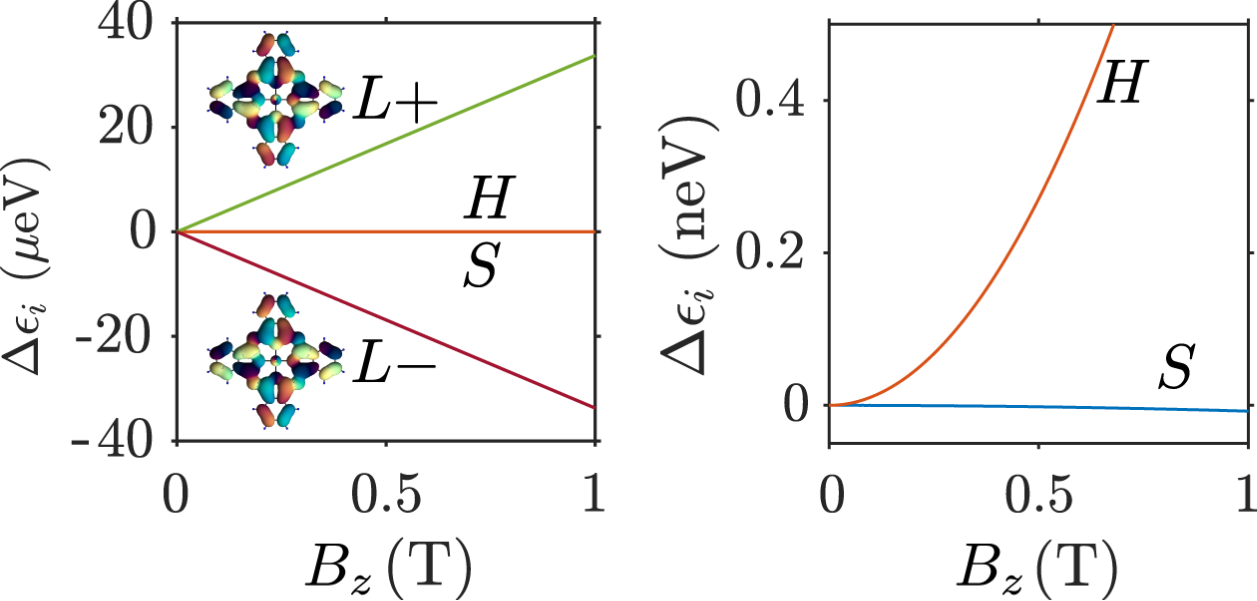
(a) Dependence of the single particle orbital energies on the magnetic field strength. From this, the effective orbital moment of the LUMOs, here depicted in their complex representation, can be extracted as μ_orb_ = 33.7 μeVT^−1^. The energies of the SOMO and HOMO orbitals depend quadratically on the magnetic field and involve a much lower scale than the LUMOs, as seen in the close-up in panel (b).

Here (*x*_α_, *y*_α_) are the in-plane atomic coordinates. Owing to the planar geometry of CuPc, 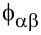 depends only on the *z*-component *B_z_* of the magnetic field **B**. In Figure 3 we show the dependence of the energies of the frontier molecular orbitals on the strength of the magnetic field in *z*-direction, *B_z_*. For the two LUMOs we observe a linear dependence on the magnetic field, yielding an effective orbital moment of μ_orb_ = 33.7 μeVT^−1^, while the LUMO−(+) goes down (up) in energy with *B_z_* (Figure 3a). The energies of the HOMO and the SOMO, however, scale quadratically with the magnetic field at a much lower scale (Figure 3b). This behaviour is expected, since the *a*_1_*_u_* and *b*_1_*_g_* representations have characters +1 under 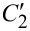 rotations, which transform *B_z_* to −*B_z_*. Thus the energies of HOMO and SOMO can not depend on the sign of *B_z_* and must move at least quadratically with *B_z_*. The two-dimensional *e_g_* representation on the other hand has zero character under 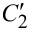 rotations, which implies that the constituents of *e_g_* transform under such rotations either with different signs or into each other; indeed under a 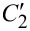 rotation LUMO+ is mapped onto LUMO− and vice versa.

Finally, the interaction of electronic spins with magnetic fields is represented by adding a Zeeman term 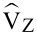 to Equation 1,

[27]
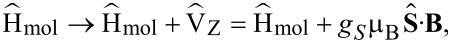


where *g_S_* = 2 and **S** is the total spin operator on the molecule written in the frontier orbital basis.

#### Effective low-energy Hamiltonian

Putting everything together, an effective low-energy Hamiltonian including magnetic interaction terms for both orbital and spin degrees of freedom can thus be given. It reads

[28]
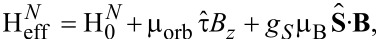


where 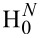 is the Hamiltonian for the corresponding low-energy *N*-particle subblock as given by Equation 12 and Equation 24.

### Dynamics and transport

#### Reduced density operator and current

The transport calculations for the molecule in an STM setup are done by using the formalism introduced in earlier works [18,26–27]. For the sake of clarity, in the following we briefly discuss the main steps to obtain the current through the molecule. The full system is described by the Hamiltonian

[29]
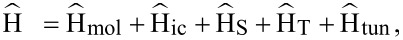


where 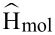 describes the isolated molecule, see Equation 1. To incorporate image charge effects in our model, leading to renormalizations of the energies of the system’s charged states [28], we included a term 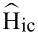
[11],

[30]
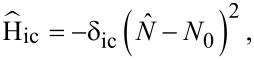


where 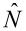 is the particle number operator on the molecule. Electrostatic considerations regarding the geometry of the STM setup yielded δ_ic_ ≈ 0.32 eV [11]. The Hamiltonians 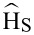 and 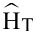 corresponding to substrate (S) and tip (T), respectively, are describing noninteracting electronic leads. They read

[31]
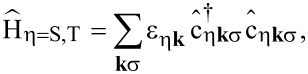


where 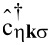 creates an electron in lead η with spin σ and momentum **k**. The tunneling Hamiltonian 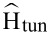 finally is given by

[32]
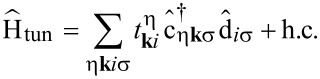


It contains the tunneling matrix elements 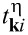, which are obtained by calculating the overlap between the lead wavefunctions 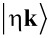 and the molecular orbitals 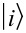
[26]. They yield the tunneling rates


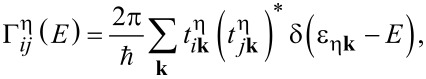


which are of the order of 10^−6^ eV and 10^−9^ eV for the substrate and the tip, respectively. Finally, the dynamics of the transport itself is calculated by evaluating the generalized master equation,

[33]
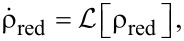


for the reduced density operator [26,29] ρ_red_ = Tr_S,T_(ρ). The Liouvillian superoperator

[34]
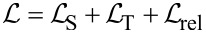


contains the terms 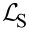 and 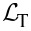 describing tunneling from and to the substrate and the tip, respectively. To account for relaxation processes leading to de-excitation of molecular excited states, we included a relaxation term 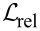, analogously to [30]:

[35]
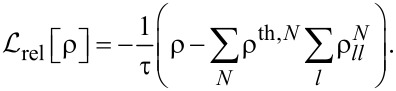


It depends on the deviation of ρ from the thermal distribution ρ^th,N^ of the *N*-particle subblock, which is given by a Boltzmann distribution:

[36]
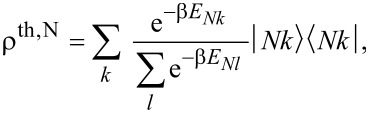


with β = (*k*_B_*T*)^−1^. Since 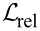 acts separately on each *N*-particle subblock, it conserves the particle number on the molecule and thus does not contribute to transport directly. In this work, the relaxation factor 1/τ is around the same order of magnitude as the mean tip tunneling rate onto the molecule. In particular, we are interested in the stationary solution 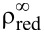 for which 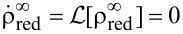. Finally, the current through the system in the stationary limit can be evaluated as

[37]
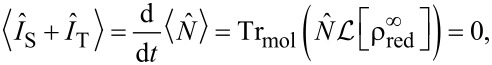


yielding the current operator for lead η as 
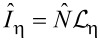
.

#### Transport characteristics

**Figure 4:**
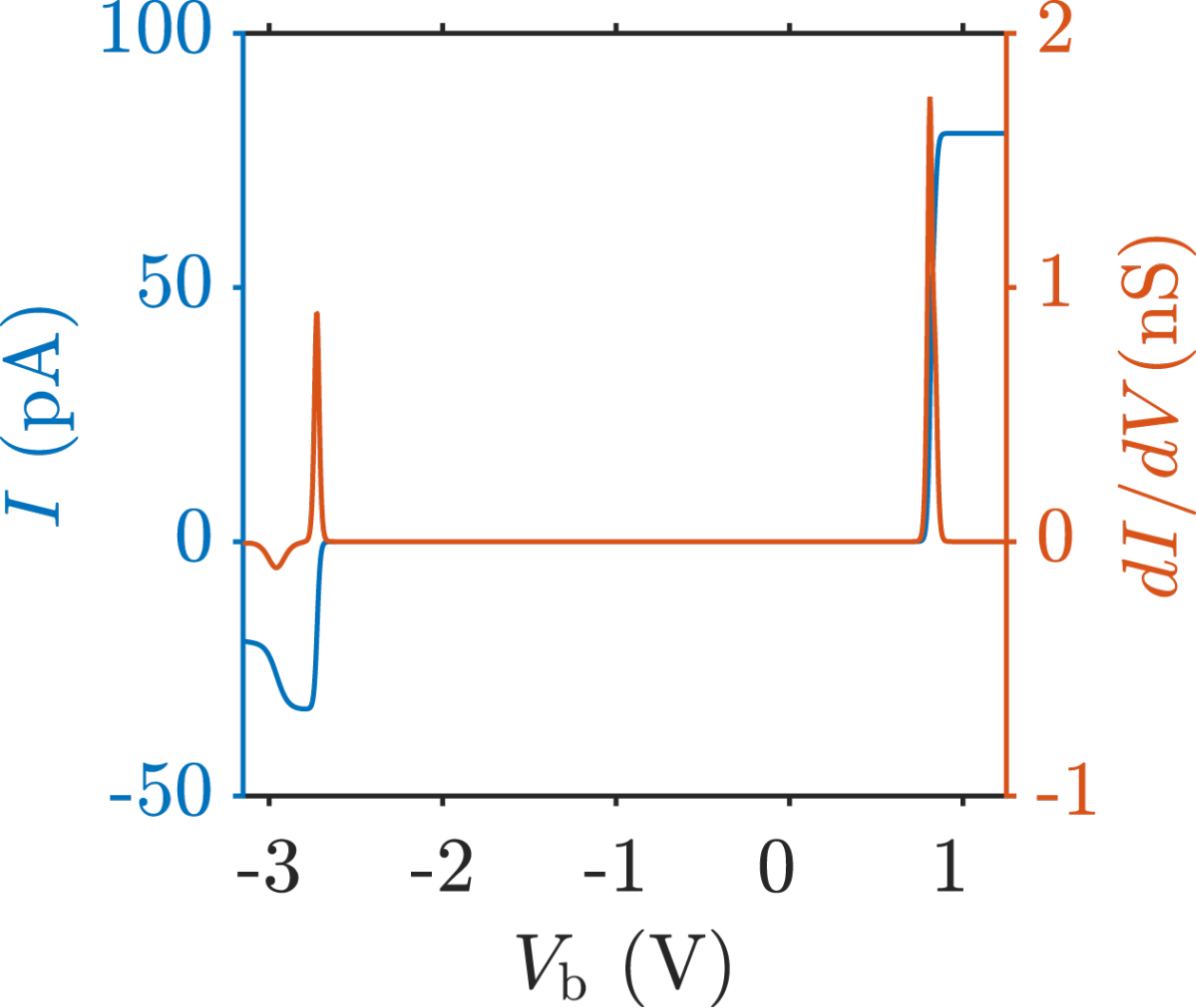
Current and differential conductance curves exhibiting the anionic (cationic) resonance at positive (negative) bias voltage. Note that in contrast to all other results in this work, this curve is taken at a temperature of 60 K to emphasize the resonances in the *dI*/*dV* curve.

In this work, a tip–molecule distance of 5 Å was used and simulations were done at the temperature *T* = 1 K. We assumed a renormalization of the single particle energies δ*_i_* = δ =1.83 eV (cf. Equation 3), an image-charge renormalization δ_ic_ = 0.32 eV and a dielectric constant ε_r_ = 2.2 in order to fit our spectrum to the experiment of Swart et al. [14], which was carried out with CuPc on a NaCl(3 ML)/Cu(100) substrate with a workfunction of 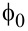 = 4.65 eV. With this, we find a triplet–singlet separation of the anionic ground state of 18 meV, which is in good agreement with experimental measurements of 21 meV [8]. Numerical results for the current and the differential conductance, according to Equation 37 and using the full Hamiltonian 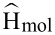 in Equation 29, are shown in Figure 4. Anionic (cationic) resonances at positive (negative) bias voltages are clearly seen.

Notice that, in our model, the bias voltage at which a tip-mediated transition from the *m*-th neutral state to the *n*-th anionic state of the molecule is happening is

[38]
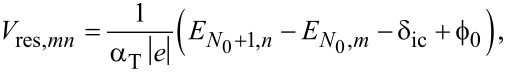


where *e* is the electron charge and α_T_ accounts for the fact that in STM setups the bias voltage drops asymmetrically across the junction. Electrostatic considerations yielded α_T_ = 0.59 for the tip and α_S_ = −0.16 for the substrate [11]. If given without indices, *V*_res_ denotes the bias voltage corresponding to the groundstate-to-groundstate resonance.

The negative differential conductance at large negative bias in Figure 4 is caused by blocking due to population of excited states of the molecule. This has already been discussed in a previous work [27] and will not be of further interest here.

#### Transport simulations at finite magnetic fields

**Figure 5:**
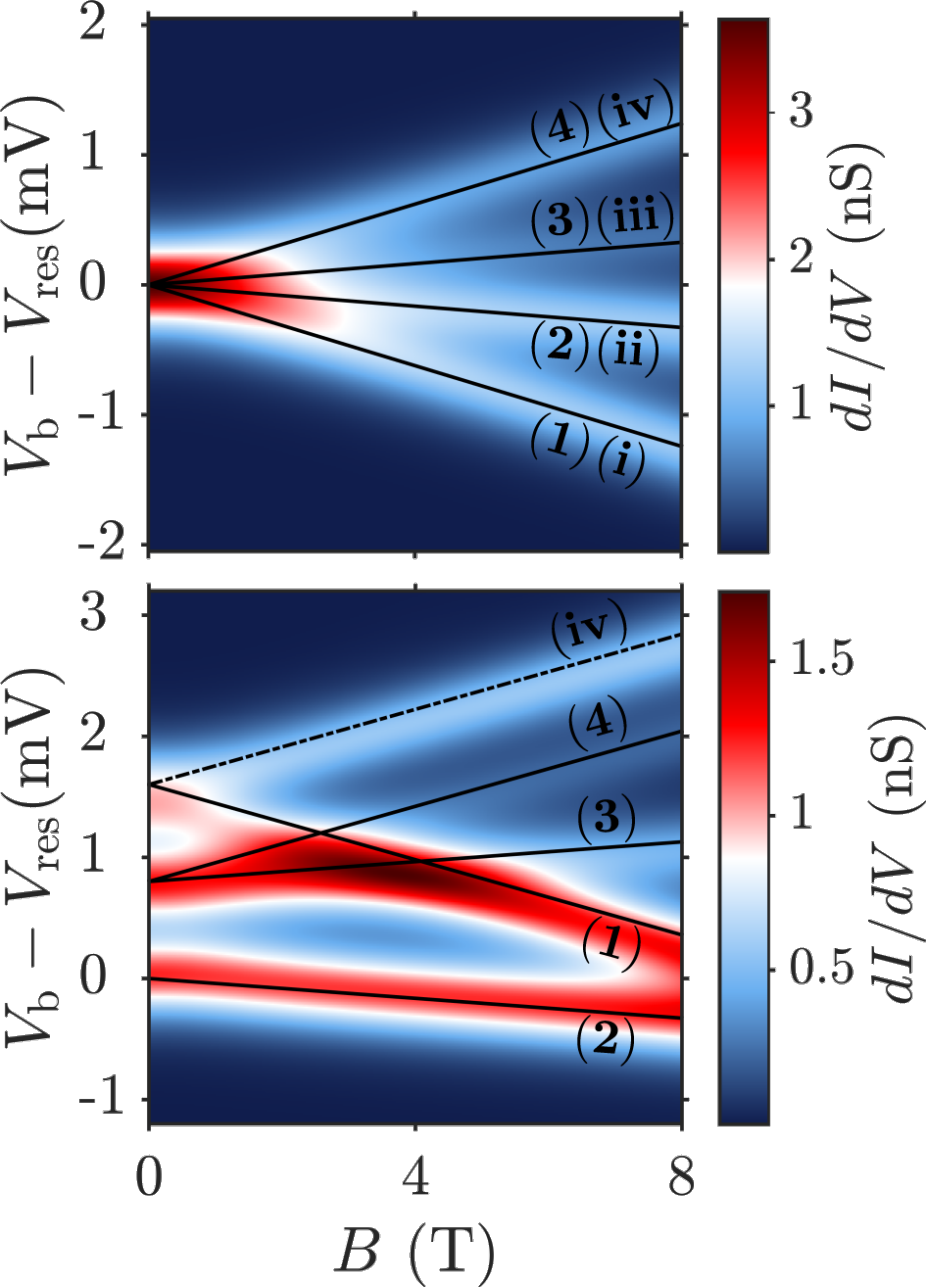
Differential conductance maps as a function of the strength *B_z_* of the magnetic field in *z*-direction. Upper (lower) panel: Spin–orbit interaction switched off (on). Solid and dashed lines depict the addition spectrum as calculated from the effective spin Hamiltonian, cf. Equation 28. Transitions starting from the neutral groundstate are denoted by solid lines, those from the neutral excited state by dashed lines.

In Figure 5 we show the splitting of the anionic resonance with applied magnetic field in a *dI*/*dV* map. In the upper panel SOI is switched off, whereas in the lower panel it is switched on. One striking difference at first glance is the zero-field splitting for non-vanishing SOI, which is proportional to λ_1_ but enhanced by the bias drop, cf. Equation 38. For vanishing SOI, when *S_z_* is a good quantum number, we can readily identify the corresponding transitions by using the effective spin Hamiltonian introduced in Equation 28. In the following, transitions from the neutral groundstate will be denoted by arabic numbers:


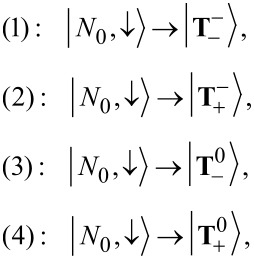


while transitions from the neutral excited state will be denoted by Roman numerals:


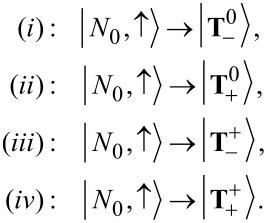


Other transitions are forbidden due to the selection rule for *S_z_*, Δ*S_z_* = ±(1/2). The reason for the splitting into four lines observed in the upper panel of Figure 5 is that the orbital moment of the LUMO is not of the same size as the Bohr magneton.

For non-vanishing SOI, see lower panel of Figure 5, the definite assignment of transitions is not straightforward, at least for small magnetic fields. Since 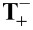 and 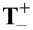 are shifted downward by SOI, transition (2) now is the lowest lying transition, whereas transition (1) is shifted upward due to the positive contribution +λ_1_ to 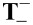. Furthermore, transition (iv) is the only excited-state transition which can be definitely assigned to a line in the lower panel in Figure 5.

**Figure 6:**
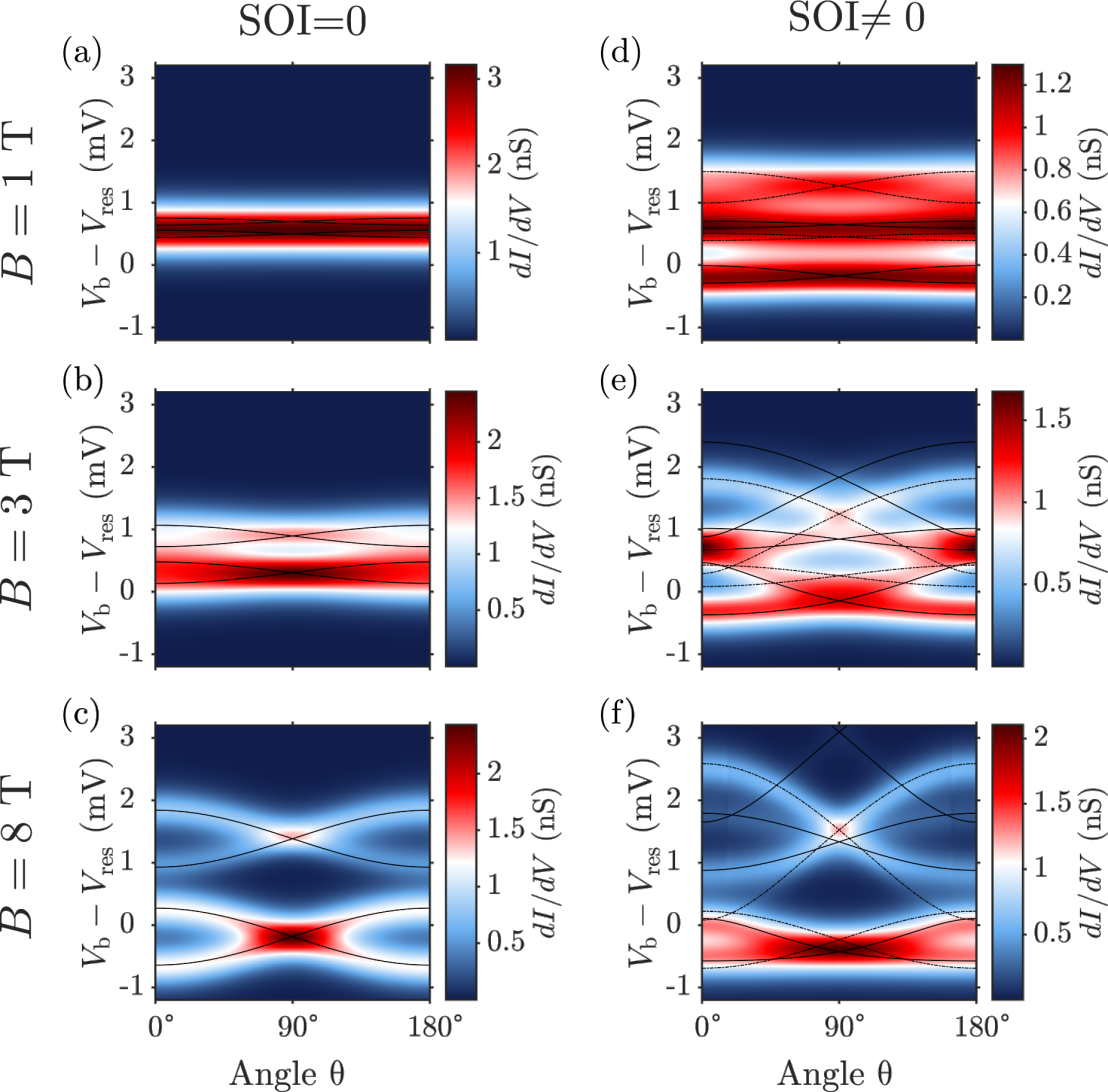
Differential conductance maps vs the angle θ, formed by the applied magnetic field with the *z*-axis. Left (right) panels are without (with) SOI. Upper, middle and lower panels are calculated for a magnetic field strength of 1, 3 and 8 T, respectively. Solid and dashed lines depict the addition spectrum as calculated from the effective spin Hamiltonian, cf. Equation 28. Transitions starting from the neutral groundstate are denoted by solid lines, those from the neutral excited state by dashed lines.

Figure 6 finally shows *dI/dV* maps as a function of the angle θ between the magnetic field and the *z*-axis. Panels (a), (b) and (c) show results obtained with vanishing SOI and panels (d), (e) and (f) are for finite SOI. Again, the results were fitted using the effective spin Hamiltonian introduced in Equation 28 with good agreement. The respective transitions can be identified by checking the assigned transitions in Figure 5 at the corresponding field strength.

Already at |**B**| = *B* = 1 T, cf. panels (a) and (d), the influence of SOI can be clearly seen. While for vanishing SOI any anisotropy of the *dI/dV* map is hidden beneath the temperature broadening, for finite SOI a slight θ-dependence can be observed. For *B* = 3 T, now also in the vanishing SOI case, Figure 6b, a slight anisotropy due to the orbital moment of the LUMOs can be observed, although still blurred by temperature. Again, at finite SOI in Figure 6e there is a much more pronounced dependence on θ. The high conductance areas at θ = 0° and θ = 180° for *V*_b_ − *V*_res_ ≈ 0.8 meV correspond to the high conductance area in the middle of Figure 5 bottom, where many transitions are taking place at the same time. At *B* = 8 T, the magnetic field is dominating and a characteristic double cosine-like behaviour of the resonances can be observed, for both the case with no SOI, Figure 6c, and finite SOI, Figure 6f. For vanishing SOI, this behaviour is caused by the orbital moment of the LUMOs, since they interchange their positions when going from *B_z_* to −*B_z_*. The overall splitting between the double cosines, most evident at θ = 90°, is caused by the Zeeman term. The results for *B* = 8 T in Figure 6f at finite SOI are similar to those in Figure 6c, with the only difference that the cosine at large biases is more stretched, the one at low bias more compressed.

## Conclusion

We established a model Hamiltonian for CuPc which accounts for electron–electron, spin–orbit and magnetic interactions in a minimal single particle basis represented by four frontier orbitals; the SOMO, the HOMO and two degenerate LUMOs. The distinct properties of these orbitals under rotations allowed us to deduce selection rules for matrix elements of the Coulomb interaction, which drastically reduce the number of nonvanishing terms and simplify the numerical diagonalization of the full many-body Hamiltonian. For the low-energy parts of the neutral and anionic blocks of the many-body spectrum we could further derive an effective spin Hamiltonian, capturing both SOI-induced splittings and magnetic anisotropy. Analogous Hamiltonians accounting for the effect of atomic SOI in molecular systems with orbital degeneracies have been derived for example in carbon nanotubes [31].

In order to study fingerprints of the SOI under realistic experimental conditions, we have studied the magnetotransport characteristics of a CuPc based junction in an STM setup. To this extent, a generalized master equation for the reduced density matrix associated to the full many-body Hamiltonian had to be solved in order to numerically obtain both the current and the differential conductance. Noticeably, by using the effective spin Hamiltonian, it was possible to reconstruct the nature of the many-body resonances observed in the numerical calculations.

In summary, we believe that our work significantly advances the present understanding of spin properties of CuPc. Moreover, the flexibility of our model Hamiltonian approach opens new perspectives for the investigation of other configurationally similar metallorganic compounds.

## Supporting Information

File 1 Transformation from the atomic to the molecular orbital basis.

File 2 Symmetries in the frontier orbitals basis.

File 3 Details on the perturbative treatment of the SOI.
