# Supplementary material for: Effects of spin–orbit coupling and many-body correlations in STM transport through copper phthalocyanine
Source: Beilstein J Nanotechnol. 2015 Dec 22;6:2452–62. doi: 10.3762/bjnano.6.254 (PMC4734453; doi:10.3762/bjnano.6.254)
Supplement: File 1 — Transformation from the atomic to the molecular orbital basis. [file Beilstein_J_Nanotechnol-06-2452-s001.pdf]

Supporting Information

for

Effects of spin–orbit coupling and many-body correlations in STM-transport through copper phthalocyanine

Benjamin Siegert*, Andrea Donarini and Milena Grifoni

Address: Institut für Theoretische Physik, Universität Regensburg, D-93040, Germany

Email: Benjamin Siegert* - benjamin.siegert@ur.de

* Corresponding author

Transformation from the atomic to the molecular orbital basis

Transformation from the atomic to the molecular orbital basis

The Hamiltonian of a molecule in Born-Oppenheimer approximation, after dismissing terms which only depend on the positions of the nuclei and are therefore constant, can be written as

$$\begin{aligned}\hat{\mathrm{H}} =& \sum_{\substack{\alpha\beta\sigma\\ mn}} \left(h_{\alpha m,\beta n} + \Delta V^{\mathrm{ion}}_{\alpha m,\beta n}\right) \hat{\mathrm{d}}^{\dagger}_{\alpha m\sigma}\hat{\mathrm{d}}_{\beta n\sigma} \\ &+\frac{1}{2}\sum_{\substack{\alpha\beta\gamma\delta\\ mnop}}\sum_{\sigma\sigma'} V^{mnop}_{\alpha\beta\gamma\delta}\,\hat{\mathrm{d}}^{\dagger}_{\alpha m\sigma}\hat{\mathrm{d}}^{\dagger}_{\gamma p\sigma'}\hat{\mathrm{d}}_{\delta q\sigma'}\hat{\mathrm{d}}_{\beta n\sigma},\end{aligned} \tag{1}$$

where $\hat{\mathrm{d}}^{\dagger}_{\alpha m\sigma}$ creates an electron in the atomic orbital $|\alpha m\sigma\rangle$ with orbital quantum number m and spin σ centered at atom α. Further we have defined

$$h_{\alpha m,\beta n} := \epsilon_{\alpha m}\delta_{\alpha\beta}\delta_{mn} + b_{\alpha m,\beta n}, \tag{2}$$

where $\epsilon_{\alpha m}$ is the energy of orbital m on atom α and $b_{\alpha m,\beta n}$ is the hopping integral between orbital m on atom α and orbital n on atom β. All non-hopping terms can be condensed in the crystal field correction

$$\Delta V^{\mathrm{ion}}_{\alpha m,\beta n} := \sum_{\gamma}^{\gamma\neq\alpha,\beta} \langle \alpha m\sigma|\hat{\mathrm{V}}_{\gamma}|\beta n\sigma\rangle\,, \tag{3}$$

where $\hat{V}_{\gamma}$ is the atomic core potential at $\mathbf{r}_{\gamma}$. Equation (3) defines the crystal field correction to the single particle Hamiltonian. Finally, we have the ordinary matrix elements $V^{mnop}_{\alpha\beta\gamma\delta}$ of the Coulomb interaction.

The $h_{\alpha m,\beta n}$ are elements of a matrix $\mathbf{h}$ which corresponds to the single particle Hamiltonian of the molecule with only onsite energies and hopping terms. After performing a transformation to the molecular orbital basis, in which $\mathbf{h}$ is diagonal, $|i\sigma\rangle = \sum_{\alpha m} c_{i\alpha m}\,|\alpha m\sigma\rangle$, and using the approximation that the basis $|\alpha m\sigma\rangle$ is orthogonal, the Hamiltonian reads:

$$\begin{aligned}\hat{\mathrm{H}} =& \sum_{ij\sigma}\left(\epsilon_i\delta_{ij} + \Delta V^{\mathrm{ion}}_{ij}\right)\hat{\mathrm{d}}^{\dagger}_{i\sigma}\hat{\mathrm{d}}_{j\sigma} \\ &+\frac{1}{2}\sum_{ijkl}\sum_{\sigma\sigma'} V_{ijkl}\,\hat{\mathrm{d}}^{\dagger}_{i\sigma}\hat{\mathrm{d}}^{\dagger}_{k\sigma'}\hat{\mathrm{d}}_{l\sigma'}\hat{\mathrm{d}}_{j\sigma},\end{aligned} \tag{4}$$

where $\Delta V^{\mathrm{ion}}_{ij}$ now is

$$\Delta V^{\mathrm{ion}}_{ij} = \sum_{\substack{\alpha\beta\\ mn}} c^{*}_{i\alpha m}c_{j\beta n}\,\Delta V^{\mathrm{ion}}_{\alpha m,\beta n}. \tag{5}$$
