# Supplementary material for: Effects of spin–orbit coupling and many-body correlations in STM transport through copper phthalocyanine
Source: Beilstein J Nanotechnol. 2015 Dec 22;6:2452–62. doi: 10.3762/bjnano.6.254 (PMC4734453; doi:10.3762/bjnano.6.254)
Supplement: File 2 — Symmetries in the frontier orbitals basis. [file Beilstein_J_Nanotechnol-06-2452-s002.pdf]

Supporting Information

for

Effects of spin–orbit coupling and many-body correlations in STM-transport through copper phthalocyanine

Benjamin Siegert*, Andrea Donarini and Milena Grifoni

Address: Institut für Theoretische Physik, Universität Regensburg, D-93040, Germany

Email: Benjamin Siegert* - benjamin.siegert@ur.de

* Corresponding author

Symmetries in the frontier orbitals basis

Symmetries in the frontier orbitals basis

One huge simplification which is possible in the molecular orbital basis is the reduction of the size of our Hilbert space $\mathcal{H}$, which occurs by retaining few relevant molecular orbitals only. To this end we split the full molecular basis into frozen and dynamic orbitals, where N_f of the frozen orbitals are assumed to be always fully occupied and the remaining N_e set to be always empty. We do not make any assumption about the occupation of the N_d dynamic states. Whether these N_d frontier orbitals are full or empty depends on the electrochemical potential of the molecule, and on whether an exchange of electrons with the environment is possible.

In the occupation number representation a general state of the Fock space then looks like

$$|\Psi\rangle \approx \underbrace{|11\dots11\rangle}_{2N_f} \otimes \underbrace{|n_{k\uparrow}n_{k\downarrow}\dots n_{l\uparrow}n_{l\downarrow}\rangle}_{2N_d} \otimes \underbrace{|00\dots00\rangle}_{2N_e}. \tag{1}$$

In this work we assume the molecule to be neutral under equilibrium conditions, with 195 valence electrons. Thus, the orbitals we choose to build up the subspace of dynamic orbitals are orbitals Nrs. 97-100, see Fig. 1 (b) in the main text. This choice results in the lowest 96 molecular orbitals being doubly filled. Note that the choice of the LUMO states $L\pm$ rather than $L_{zx/yz}$ is convenient due to the fact that these orbitals acquire a definite phase upon rotations of 90 degrees around the main symmetry axis of the molecule. Specifically, for the four frontier orbitals S, H and $L\pm$, the acquired phases are $\phi_S = \pi$, $\phi_H = 0$ and $\phi_{L\pm} = \pm\frac{\pi}{2}$, respectively. This in turn imposes symmetry constraints on the Hamiltonian. Consider e.g. the Coulomb interaction

$$V_{ijkl} = \frac{1}{4\pi\varepsilon_0}\iint \mathrm{d}^3r_1\,\mathrm{d}^3r_2\,\psi_i^*(\mathbf{r}_1)\psi_j(\mathbf{r}_1)\frac{1}{|\mathbf{r}_1-\mathbf{r}_2|}\psi_k^*(\mathbf{r}_2)\psi_l(\mathbf{r}_2). \tag{2}$$

Then, in the frontier orbital basis it holds that:

$$V_{ijkl} = \mathrm{e}^{-i(\phi_i-\phi_j+\phi_k-\phi_l)}\,V_{ijkl}. \tag{3}$$

Therefore a given matrix element of the Coulomb interaction V_{ijkl} is different from zero only if the sum of the corresponding phases adds up to multiples of 2π: $\phi_i - \phi_j + \phi_k - \phi_l = 2\pi \cdot n,\ n \in \mathbb{Z}$. In Tab. 1 we list all nonvanishing matrix elements of the Coulomb interaction which are used in this work. For the crystal field correction $\Delta V_{ij}^{\mathrm{ion}}$ it can be shown that:

$$\Delta V_{ij}^{\mathrm{ion}} = \mathrm{e}^{-i(\phi_i-\phi_j)}\,\Delta V_{ij}^{\mathrm{ion}} \tag{4}$$

$$\Rightarrow \Delta V_{ij}^{\mathrm{ion}} = \Delta V_{ii}^{\mathrm{ion}}\,\delta_{ij}, \tag{5}$$

since all phases ϕ_i are different; $\phi_i \neq \phi_j$ for $i \neq j$. Hence $\Delta V_{ij}^{\mathrm{ion}}$ is diagonal in the $\{S,H,L\pm\}$ basis. In the following we treat the $\Delta V_{ii}^{\mathrm{ion}}$ as free parameters and include them in the paramteter δ_i entering Eq. 3 in the main text.

U_S	11.352 eV	$J^{\mathrm{ex}}_{HL} = -\tilde{J}^{\mathrm{p}}_{HL+L-}$	548 meV
U_H	1.752 eV	J^{ex}_{L+L-}	258 meV
$U_L = U_{L+L-}$	1.808 eV	J^{p}_{L+L-}	168 meV
U_{SH}	1.777 eV	$J^{\mathrm{ex}}_{SL} = -\tilde{J}^{\mathrm{p}}_{SL+L-}$	9 meV
U_{SL}	1.993 eV	$J^{\mathrm{ex}}_{SH} = J^{\mathrm{p}}_{SH}$	2 meV
U_{HL}	1.758 eV		

Table 1: Major nonvanishing Coulomb integrals between the SOMO(S), the HOMO(H), the LUMO+ and the LUMO−. When the LUMOs need to be distinguished, they are denoted as $L+$ or $L-$, otherwise just by L. All values are calculated numerically using Monte Carlo integration [1] of the real space orbitals and renormalized by a constant $\epsilon_r = 2.2$.

References

[1] M. Galassi et al., *GNU Scientific Library Reference Manual*, 3rd ed., https://www.gnu.org/software/gsl/manual/
