# Supplementary material for: Effects of spin–orbit coupling and many-body correlations in STM transport through copper phthalocyanine
Source: Beilstein J Nanotechnol. 2015 Dec 22;6:2452–62. doi: 10.3762/bjnano.6.254 (PMC4734453; doi:10.3762/bjnano.6.254)
Supplement: File 3 — Details on the perturbative treatment of the SOI. [file Beilstein_J_Nanotechnol-06-2452-s003.pdf]

Supporting Information

for

Effects of spin–orbit coupling and many-body correlations in STM-transport through copper phthalocyanine

Benjamin Siegert*, Andrea Donarini and Milena Grifoni

Address: Institut für Theoretische Physik, Universität Regensburg, D-93040, Germany

Email: Benjamin Siegert* - benjamin.siegert@ur.de

* Corresponding author

Details on the perturbative treatment of the SOI

Details on the perturbative treatment of the SOI

In addition to the states introduced in Eq. 14 in the main text, the following states must be also taken into account when performing second order perturbation theory:

$$
\begin{aligned}
|L\tau\uparrow, L\tau\downarrow\rangle &= \hat{\mathrm{d}}^{\dagger}_{L\tau\uparrow}\hat{\mathrm{d}}^{\dagger}_{L\tau\downarrow}|\Omega\rangle\,, \\
|L\tau\sigma, L\bar{\tau}\sigma'\rangle &= \hat{\mathrm{d}}^{\dagger}_{L\tau\sigma}\hat{\mathrm{d}}^{\dagger}_{L\tau\sigma'}|\Omega\rangle\,, \\
|S\uparrow, S\downarrow\rangle &= \hat{\mathrm{d}}^{\dagger}_{S\uparrow}\hat{\mathrm{d}}^{\dagger}_{S\downarrow}|\Omega\rangle\,,
\end{aligned}
\tag{1}
$$

with $E_{L\tau\uparrow,L\tau\downarrow} = E_{L\tau\sigma,L\bar{\tau}\sigma'} = \Delta_1$ and $E_{S\uparrow,S\downarrow} = \Delta_2$. In the basis introduced in Eqs. 14 in the main text and Eq. (1), $\hat{\mathrm{V}}_{\mathrm{SO}}$ is blockdiagonal and decomposes into six subblocks: two three-dimensional, two two-dimensional, one four-dimensional and one one-dimensional subblocks.

The four dimensional subblock describes the effects of SOI on the $\mathbf{T}^+_+$ and $\mathbf{T}^-_-$ states. Written in the basis $\{|\mathbf{T}^+_+\rangle, |\mathbf{T}^-_-\rangle, |L^+\uparrow, L^-\downarrow\rangle, |S\uparrow, S\downarrow\rangle\}$, the Hamiltonian reads

$$
\begin{aligned}
H = &\begin{pmatrix} -J^{\mathrm{ex}}_{SL} & 0 & 0 & 0 \\ 0 & -J^{\mathrm{ex}}_{SL} & 0 & 0 \\ 0 & 0 & \Delta_1 & 0 \\ 0 & 0 & 0 & \Delta_2 \end{pmatrix} \\
&+ \begin{pmatrix} \lambda_1 & 0 & -\sqrt{2}\lambda_2 & \sqrt{2}\lambda_2 \\ 0 & \lambda_1 & \sqrt{2}\lambda_2 & -\sqrt{2}\lambda_2 \\ -\sqrt{2}\lambda_2 & \sqrt{2}\lambda_2 & \lambda_1 & 0 \\ \sqrt{2}\lambda_2 & -\sqrt{2}\lambda_2 & 0 & 0 \end{pmatrix}.
\end{aligned}
\tag{2}
$$

The degeneracy of the unperturbed states $\mathbf{T}^+_+$ and $\mathbf{T}^-_-$ and the fact that there are no matrix-elements which couple these states require the use of second order degenerate perturbation theory. Applying it yields the following matrix M:

$$
M = A \cdot \begin{pmatrix} 1 & -1 \\ -1 & 1 \end{pmatrix}, \tag{3}
$$

where the prefactor A is given by

$$
A = -2\lambda_2^2 \left(\frac{1}{\Delta_1 + J^{\mathrm{ex}}_{SL}} + \frac{1}{\Delta_2 + J^{\mathrm{ex}}_{SL}} \right). \tag{4}
$$

Diagonalization of M gives the second-order energy corrections

$$
\Delta E(\alpha) = \lambda_1, \tag{5}
$$

$$
\Delta E(\beta) = \lambda_1 - 4\lambda_2^2 \left(\frac{1}{\Delta_1 + J^{\mathrm{ex}}_{SL}} + \frac{1}{\Delta_2 + J^{\mathrm{ex}}_{SL}} \right), \tag{6}
$$

and the correct linear combinations of the states $\mathbf{T}_+^+$ and $\mathbf{T}_-^-$:

$$|\alpha\rangle = \frac{1}{\sqrt{2}}\Big(|\mathbf{T}_+^+\rangle + |\mathbf{T}_-^-\rangle \Big) \tag{7}$$

$$|\beta\rangle = \frac{1}{\sqrt{2}}\Big(|\mathbf{T}_+^+\rangle - |\mathbf{T}_-^-\rangle \Big). \tag{8}$$

Writing H in the basis $\{|\alpha\rangle, |\beta\rangle, |L^+\uparrow, L^-\downarrow\rangle, |S\uparrow, S\downarrow\rangle\}$ yields:

$$\tilde{H} = \begin{pmatrix} -J_{SL}^{\text{ex}} & 0 & 0 & 0 \\ 0 & -J_{SL}^{\text{ex}} & 0 & 0 \\ 0 & 0 & \Delta_1 & 0 \\ 0 & 0 & 0 & \Delta_2 \end{pmatrix} + \begin{pmatrix} \lambda_1 & 0 & 0 & 0 \\ 0 & \lambda_1 & -2\lambda_2 & 2\lambda_2 \\ 0 & -2\lambda_2 & \lambda_1 & 0 \\ 0 & 2\lambda_2 & 0 & 0 \end{pmatrix}. \tag{9}$$

We see that $|\alpha\rangle$ stays unaffected by the perturbation, whereas $|\beta\rangle$ will change:

$$|\beta\rangle \rightarrow |\beta\rangle + 2\,\frac{\lambda_2}{\Delta_1 + J_{SL}^{\text{ex}}}\,|L^+\uparrow, L^-\downarrow\rangle - 2\,\frac{\lambda_2}{\Delta_2 + J_{SL}^{\text{ex}}}\,|S\uparrow, S\downarrow\rangle\,. \tag{10}$$

The mixing of $\mathbf{T}_+^-$ and $\mathbf{T}_-^+$ is caused by a pair-hopping term in the Hamiltonian, more precisely by

$$\frac{1}{2}J_{L+L-}^{\text{p}} \sum_\sigma \Big(\hat{\text{d}}_{L+\sigma}^\dagger \hat{\text{d}}_{L+\bar{\sigma}}^\dagger \hat{\text{d}}_{L-\bar{\sigma}} \hat{\text{d}}_{L-\sigma} + \text{h.c.} \Big)\,, \tag{11}$$

which couples $\mathbf{T}_+^-$ and $\mathbf{T}_-^+$ to the following states:

$$|a\rangle = \frac{1}{\sqrt{2}} \hat{\text{d}}_{H\uparrow}^\dagger \hat{\text{d}}_{H\downarrow}^\dagger \Big(\hat{\text{d}}_{L+\uparrow}^\dagger \hat{\text{d}}_{L+\downarrow}^\dagger - \hat{\text{d}}_{L-\uparrow}^\dagger \hat{\text{d}}_{L-\downarrow}^\dagger \Big) |0\rangle\,,$$

$$|b\rangle = \frac{1}{\sqrt{2}} \hat{\text{d}}_{H\uparrow}^\dagger \hat{\text{d}}_{H\downarrow}^\dagger \Big(\hat{\text{d}}_{L+\uparrow}^\dagger \hat{\text{d}}_{L+\downarrow}^\dagger + \hat{\text{d}}_{L-\uparrow}^\dagger \hat{\text{d}}_{L-\downarrow}^\dagger \Big) |0\rangle\,, \tag{12}$$

with corresponding energies E_a and $E_b = E_a + 2J_{L+L-}^{\text{p}}$. Then, after introducing

$$|\mathbf{T}_1\rangle = \frac{1}{\sqrt{2}} \left(|\mathbf{T}_+^-\rangle + |\mathbf{T}_-^+\rangle \right),$$

$$|\mathbf{T}_2\rangle = \frac{1}{\sqrt{2}} \left(|\mathbf{T}_+^-\rangle - |\mathbf{T}_-^+\rangle \right), \tag{13}$$

the Hamiltonian in the basis of these four states can be written as

$$H = \begin{pmatrix} H_{1b} & 0 \\ 0 & H_{2a} \end{pmatrix}, \tag{14}$$

with

$$H_{1b} = \begin{pmatrix} -J_{SL}^{\text{ex}} - \lambda_1 & \lambda_2 \\ \lambda_2 & E_b \end{pmatrix} \tag{15}$$

and

$$H_{2a} = \begin{pmatrix} -J_{SL}^{\text{ex}} - \lambda_1 & \lambda_2 \\ \lambda_2 & E_a \end{pmatrix}. \tag{16}$$

Diagonalization finally yields the four states

$$\begin{aligned} |1\rangle &= \frac{1}{\sqrt{1-\gamma_b^2}} \left(|\mathbf{T}_1\rangle + \gamma_b \, |b\rangle \right), \\ |2\rangle &= \frac{1}{\sqrt{1-\gamma_a^2}} \left(|\mathbf{T}_2\rangle + \gamma_a \, |a\rangle \right), \\ |\tilde{1}\rangle &= \frac{1}{\sqrt{1-\gamma_b^2}} \left(|b\rangle - \gamma_b \, |\mathbf{T}_1\rangle \right), \\ |\tilde{2}\rangle &= \frac{1}{\sqrt{1-\gamma_a^2}} \left(|a\rangle - \gamma_a \, |\mathbf{T}_2\rangle \right), \end{aligned} \tag{17}$$

with the admixture $\gamma_{a/b} \approx \frac{-\lambda_2}{E_{a/b}+J_{SL}^{\text{ex}}}$. Their energies are approximately

$$\begin{aligned} E_1 &\approx -\lambda_1 - \frac{\lambda_2^2}{E_b + J_{SL}^{\text{ex}} + \lambda_1}, \\ E_2 &\approx -\lambda_1 - \frac{\lambda_2^2}{E_a + J_{SL}^{\text{ex}} + \lambda_1}, \\ E_{\tilde{1}} &\approx E_b + \frac{\lambda_2^2}{E_b + J_{SL}^{\text{ex}} + \lambda_1}, \\ E_{\tilde{2}} &\approx E_a + \frac{\lambda_2^2}{E_a + J_{SL}^{\text{ex}} + \lambda_1}. \end{aligned} \tag{18}$$

This analysis reproduces mixing and energy splittings consistent with our numerical calculations.
